# Correction of Gummy Smile: An Essential Procedure in Facial Aesthetics

**DOI:** 10.1055/s-0044-1801265

**Published:** 2024-12-27

**Authors:** Dinesh Kadam

**Affiliations:** 1 Department of Plastic and Reconstructive Surgery, A.J. Institute of Medical Sciences and Research Centre, Mangalore, Karnataka, India

A gummy smile or high gingival smile is characterized by excessive gingiva exposure above 3 mm while smiling. A thin line of gingival exposure with a full smile is typical, particularly in females. About 14% of women and 6% of men present with excessive gingival show, which is more common in younger ages. 1 As age progresses, the upper lip drops due to decreased muscle tone. Young adults can experience significant psychological stress and anxiety due to an unpleasant smile. It is imperative for aesthetic plastic surgeons to understand this condition when considering volumizing the lips with fillers.

The underlying causes are multifactorial, arising from skeletal, dental, and soft tissue anomalies, such as vertical maxillary excess, short upper lip, hypermobile upper lip, dentoalveolar asymmetry, occlusal cant, etc. The gingival hypertrophy may result from acute or chronic inflammation or drug-induced effects. The gingival show varies in severity from mild (2–4 mm) to moderate (4–8 mm) to severe (>8 mm). 2 Many of these can be evaluated clinically; more severe ones require correction for the underlying cause with orthodontic and orthognathic procedures. However, simpler soft tissue corrections can be achieved by gingival resection and lip repositioning. I wish to highlight this procedure by illustrating a young patient seeking improvement in her smile.

A 23-year-old woman with an excessive gingival show while smiling was evaluated ( Fig. 1A ). The orthopantomography (OPG) and lateral cephalogram evaluation was done, which did not warrant any invasive bony procedures. She had a 4-mm show of gingiva on measuring the distance between the apex of the visible tooth and the lower border of the upper lip on a full smile. She had a narrow and smaller tooth crown visibility due to excessive gingiva. Both gingival resection of 4 mm and lip repositioning with sulcus excision of 8 mm (double the width of the excised gingiva) were planned ( Fig. 2A–D ). The procedure was performed under local anesthesia, with 1:100,000 adrenaline infiltration. The gingival resection extended from the premolar to the premolar tooth of the upper alveolus. The sulcus incision was sutured with a 5–0 absorbable suture, and the patient was discharged following the procedure. The postoperative course was uneventful. At 3 months of follow-up, the patient was satisfied with her natural smile ( Fig. 1B ).

**Fig. 1:**
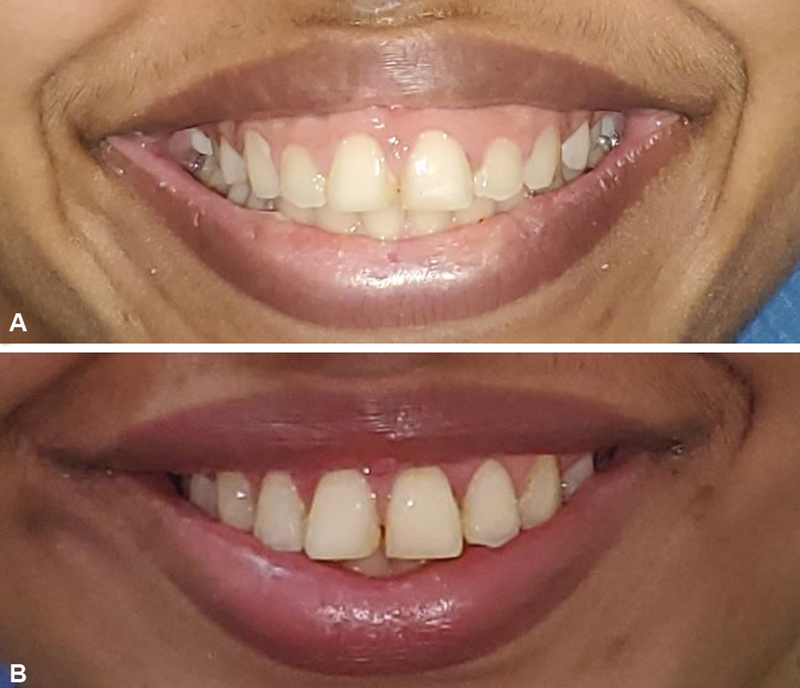
( **A** ) Gummy smile on presentation. ( **B** ) At 3 months of follow-up.

**Fig. 2:**
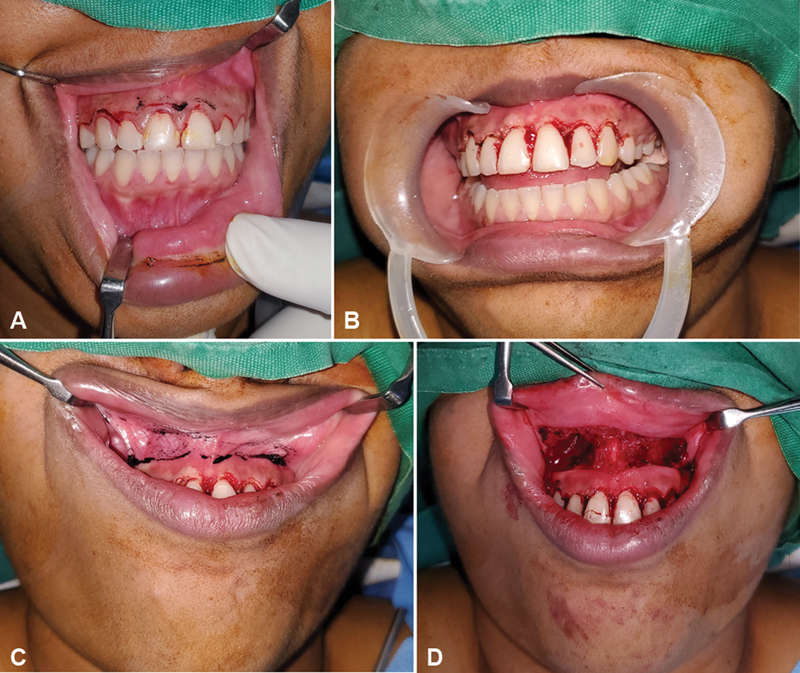
( **A** ) Marking of gingival resection, ( **B** ) resection of gingiva 4 mm with greater teeth visibility, ( **C** ) marking of resection of 8-mm sulcus, and ( **D** ) raw area closed with 5–0 sutures.

The procedure is well described in the dental literature and offers a less invasive alternative to the otherwise extensive orthognathic procedures in select cases. 2 Technological innovations have made the procedure even more precise and safer, with the utility of lasers and digital planning. 3 Botox injections have also been used for a hypermobile lip with more than 30% retraction during the smile. This procedure is useful for plastic surgeons in a holistic approach to the aesthetic restoration of lips and smiles.
